# Study on the Relationship between the Use of Bisphosphonates for Antiosteoporosis and Vertebral Re-Fracture after Vertebroplasty

**DOI:** 10.1155/2022/3223437

**Published:** 2022-09-23

**Authors:** Li Qian, Qian Chen, Dashou Wang, Qi Pan, Qianhong Jian, Yinghong Ma

**Affiliations:** Department of Pain Medicine, Orthopedic Hospital of Guizhou Province, Guiyang 550002, Guizhou, China

## Abstract

**Objective:**

To explore the effect of bisphosphonates after vertebroplasty in patients with osteoporotic vertebral compression fractures (OVCF), and to analyze the relationship between the use of bisphosphonates and vertebral refracture.

**Methods:**

A total of 150 patients with OVCF were selected from the pain department of our hospital from January 2018 to May 2020. All patients received vertebroplasty after admission, and were divided into the surgery group (62 cases) and combined with the bisphosphonates group (combined group, 88 cases) according to whether patients had used bisphosphonates after surgery. Before surgery, 1 month, 3 months, 6 months, and 1 year after surgery, visual analogue scale (VAS), Oswestry disability index (ODI), vertebral body and femoral neck bone mineral density (BMD), and Cobb Angle were collected, and the differences among groups were compared to analyze the treatment effect. After the follow-up, patients were divided into two groups according to whether vertebral refracture occurred during the follow-up period. Clinical characteristics, general information, and surgical indicators of patients in the two groups were collected, and related factors of postoperative vertebral refracture were analyzed.

**Results:**

There were no significant differences in preoperative VAS score, ODI index, BMD value, and Cobb angle between the two groups (*P* > 0.05). At 12 months after surgery, VAS score, ODI index, and Cobb angle decreased, while BMD value increased in both groups. The VAS score, ODI index, and Cobb angle in the combined group were lower than those in the operation group, while the BMD value was higher than that in the operation group, and the difference was significant (*P* < 0.05). The results of multivariate regression analysis showed that in BMD, no postoperative antiosteoporosis treatment, bone cement leakage, and poor cement diffusion were independent risk factors for vertebral refracture after vertebroplasty in patients with vertebral compression fractures.

**Conclusion:**

In order to avoid recurrent fractures in OVCF patients, attention should be paid to BMD, whether patients take antiosteoporosis drugs, whether bone cement permeation occurs and the diffusion of bone cement, etc. The above factors are the main influencing factors leading to recurrent fractures after PKP and PVP in the clinic.

## 1. Introduction

Osteoporotic fractures are the most common bone diseases in middle-aged and elderly people, among which the spine is the most common site of osteoporotic fractures [1]. Osteoporotic vertebral compression fracture (OVCF) is one of the most common and serious complications of osteoporosis. It mostly occurs in the lower thoracic and upper lumbar segments, and the main symptoms of patients with OVCF are severe pain in the lower back, especially when changing body position [2, 3]. The treatment of OVCF varies, and most patients can obtain good clinical results with conservative treatment, while a small proportion of patients have poor results with conservative treatment and require surgical intervention. Percutaneous vertebro plasty (PVP) or percutanouskyphoplasty (PKP) are the main procedures for the current clinical treatment of OVCF. [4]. PVP and PKP, as relatively mature minimally invasive surgeries for elderly OVCF, have been widely applied in clinical practice with their advantages such as rapid relief of patients' pain symptoms, improvement of vertebral stability, reduction of bed time and early return to normal activities compared with conservative treatment [5, 6].

Although vertebral plasty in the treatment of vertebral compression fractures, but not fundamentally in the treatment of osteoporosis, in clinical practice, so there will be a part of after surgery in patients with osteoporosis will still appear the symptom of back pain, and vertebral bone cement injection, as a result of the surgery stiffness enhancement, it will lead to increased risk of vertebral fracture again [7]. At present, according to literature research findings [8, 9], most of the vertebral fractures that occur after vertebroplasty occur in the adjacent vertebrae near the operating vertebrae. At present there is no large-scale clinical study to reduce PKP holds the vertebral fractures of postoperative recurrence of related research, and the specific reasons for nonsurgical vertebral fractures also has no unified conclusion, but for postmenopausal women with osteoporosis, measures for preventing nonoperative vertebral fracture again hair, to reduce the risk of vertebral fracture again hair is of great significance. Bisphosphonates are currently the first-line drugs for the treatment of osteoporosis, which can inhibit the function of osteoclasts and induce apoptosis of osteoclasts, reduce bone resorption and thus improve bone mineral density and reduce the incidence of fractures [10–12]. There are only studies on the efficacy of vertebroplasty combined with bisphosphonate use in the treatment of OVCF, but no studies on the correlation between antiosteoporosis treatment with bisphosphonates and refracture. Therefore, this project aims to investigate the correlation between the use of bisphosphonates against osteoporosis in patients with osteoporosis after vertebroplasty on the reoccurrence of new vertebral fractures, to understand whether the use of bisphosphonates is effective in reducing the reoccurrence of vertebral fractures and to provide more definite scientific evidence to the clinic.

## 2. Clinical Data

### 2.1. Subjects

In this study, OVCF patients admitted to the Department of Pain (Guizhou Orthopedic Hospital) of our hospital from January 2018 to May 2020 with concurrent surgical treatment were collected. The clinical data of 150 patients who met the inclusion criteria were retrospectively analyzed during 2 years of postoperative follow-up. Patients were divided into the operation group and the combined group according to whether they were treated with zoledronic acid after operation.

### 2.2. Diagnostic Criteria of OVCF

Diagnosis of OVCF [13] required the combination of patient history, clinical manifestations, and imaging evidence as the judgment criteria, of which imaging examination was the main means of diagnosis and the gold standard. The diagnosis should meet the following conditions: (1) low back pain, accompanied by limited movement, the pain was aggravated when changing the body position, and the symptoms were relieved when braking rest and bed rest; (2) Typical physical examination: patients were often passive because of pain, the corresponding vertebral spinous process, and paravertebral tenderness; (3) Anteroposterial-lateral radiographs of thoracolumbar spine or CT could suggest vertebral compression and wedge degeneration, MRI of thoracolumbar spine could suggest corresponding vertebral edema, and *T* value of orthotopic spine or femoral neck BMD was less than −2.5.

### 2.3. Inclusion Criteria

OVCF patients were admitted to our hospital and emergency department. In the first diagnosis and discovery of vertebral compression fracture, vertebral compression was not more than 2 segments. The clinical data, imaging data, and follow-up data of the patients were complete.

### 2.4. Exclusion Criteria

Abnormal coagulation function; infection of the intended puncture site; vertebral compression fractures of more than 2 segments; vertebral bone protrusion into the spinal canal leads to spinal canal stenosis; spinal tumor, spinal *tuberculosis*; serious heart and lung diseases, liver and kidney dysfunction; severe gastrointestinal diseases, mental diseases; illiterate; and patients who had been treated with bisphosphonates for osteoporosis.

### 2.5. Shedding or Rejection Criteria

Duration of hospitalization in pain department <7 days; lost to follow-up after discharge; automatically discharged from hospital during treatment; serious complications occurred during hospitalization (malignant arrhythmia, myocardial infarction, cardiac arrest, local anesthetic poisoning, drug allergy, severe decline in muscle strength, etc.); those who were assessed to need surgery; and reject the experimenter midway.

### 2.6. Criteria for Loss of Follow-Up

Those who left the hospital automatically and did not have telephone contact information, those who refused to follow-up by telephone, or those who could not receive follow-up visits during the follow-up period due to death or other force majeure factors.

## 3. Treatment Process

### 3.1. Basic Treatment

The patient rested in bed, wore a belt, could be given nonsteroidal drugs to control symptoms, and daily routine pain department treatment, i.e., the appropriate phase of posterior spinal nerve block, was performed to observe the changes in the patient's pain.

### 3.2. Surgical Treatment

250 ml of 0.9% sodium chloride + 5 mg of dexamethasone intravenous drops were routinely given before surgery to prevent intraoperative adverse reactions, and SPO_2_, BP, and ECG were continuously monitored intraoperatively. The patient was placed prone on the operating table with conventional disinfection cloth, and the body surface projection of bilateral vertebral pedicle was taken under the guidance of C-arm fluoroscopy, i.e., (Bull's eye sign) as the puncture point. 3 ml of 2% lidocaine was given at the puncture point. After the effect of local layer by layer anesthesia, a bone cement puncture needle (2.5 mm) was inserted at the puncture point on both sides, and the needle was directly pierced to the pedicle of the vertebral body under c-arm anteroposition and lateral fluoroscopy monitoring. After the tip was broken, it was estimated that the tip could enter the vertebral body through the pedicle under C-arm anteroposial fluoroscopy monitoring, so the tip was slowly advanced under the guidance of C-arm anteroposial fluoroscopy to adjust the tip direction and straight into the middle 1/3 of the anterior vertebral body. C-arm anteroposial fluoroscopy suggested that the tip was in a good position. The bone cement paste was injected with a bone cement injector under the guidance of C-arm fluoroscopy, and the amount of bone cement injected into each side of each vertebral body was about 2.0 ml. Dynamic observation showed no external leakage of bone cement. The film was saved before and after needle extraction, followed by a band-aid to protect the puncture site.

### 3.3. Drug Therapy

Antiosteoporosis drugs were bisphosphonates, 70 mg of alendronate was given orally once a week or 5 mg of zoledronate was given intravenously once a year, combined with oral calcium as the basic treatment of antiosteoporosis.

## 4. Observation Methods

Outpatient follow-up or telephone follow-up were performed at 1, 3, 6, 12, and 24 months after Plo to observe the patients' recovery and recurrence of vertebral fractures. The evaluation criteria for recurrence fractures were based on the diagnosis, inclusion, and exclusion criteria of the enrolled patients, all of which were due to fracture recurrence caused by osteoporosis. The assessment measures included Oswestry disability index (ODI), bone mineral density (BMD), Visual Analogue Scale (VAS), and imaging. After the follow-up, patients were divided into two groups according to whether vertebral refracture occurred during the follow-up period. Clinical characteristics (site of initial fracture, standardized antiosteoporosis treatment), general information (age, gender, and BMI), and surgical indicators (preoperative BMD, bone cement injection amount, bone cement leakage, bone cement diffusion, etc.) of patients in the two groups were collected, and related factors of postoperative vertebral refracture were analyzed.

### 4.1. ODI

The [14] method was composed of 10 questions, including pain intensity, self-care, lifting, walking, sitting, standing, sleep interference, sexual life, social life, and travel. Each question consists of six choices. Each question consisted of 6 options, each question consisted of 5 questions with a score of 0 for the first option selected and 5 questions with a score of 5 for the 5^th^ option selected in order. If all 10 questions were answered, ODI = actual score/50(highest possible score)×100%, and 2 questions were not answered, ODI = actual score/40 × 100%. The lower the score, the less severe the dysfunction, and vice versa, the higher the score.

### 4.2. VAS Score

The [15] ruler with pain score scale was turned away from the patient, and the position representing pain degree was marked on the ruler. According to the position score, 0∼2 was classified as excellent, 3∼5 as good, 6∼8 as acceptable, and above 8 as poor. VAS score can make objective evaluation of pain, simple and easy to operate.

### 4.3. BMD Measurement

The BMD of L2–L4 vertebral body was measured and the corresponding *T* value was calculated by the osteocore-3two-dimensional cone flash full digital dual energy X-ray (DEXA) osteometer (MEDILINK Company). *T* value ≥ −1 was normal, between −2.5 and −1.0 for bone loss, and ≤−2.5 for osteoporosis.

### 4.4. Measurement of the Vertebral Kyphosis Angle (Cobb angle) at the Sagittal Position of the Fractured Vertebral Body

The vertebral body with compression fracture was confirmed on the thoracolumbar lateral X-ray film, and a horizontal line was drawn along the lower edge of the upper vertebral body and the upper edge of the lower vertebral body, respectively. The angle included by the vertical line of the two horizontal lines was the Cobb angle.

## 5. Statistical Methods

The data were input into SPSS L 6.0 for statistical analysis. Comparison was performed by *T* test, measurement data were expressed by mean soil standard deviation (Mean ± SD), count data were expressed by rate (%) using *χ*^2^ tests, and grade data were compared by rank sum test (Wilcoxon two-sample comparison method). *P* < 0.05 indicated statistically significant differences between the sample groups.

## 6. The Results

### 6.1. Comparison of General Data at Diagnosis between the Surgery Group and the Combined Group

Comparison of the general data of patients at diagnosis showed that there were no statistically significant differences between the surgery group and the combined group in the general data at diagnosis, including gender, age, BMI, BMD value, VAS score, and fracture site (*P* > 0.05). The results are shown in Table 1:

### 6.2. Comparison of Clinical Efficacy between the Operation Group and the Combined Group

**Figure 1:**
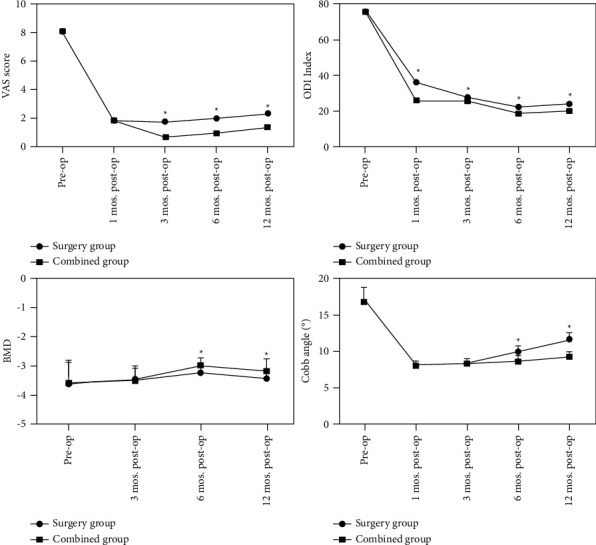
Comparison of clinical efficacy between the operation group and the combined group. Note: ^*∗*^ in the figure indicates that at this time point, the difference between the two groups is statistically significant, *P* < 0.05.

There were no significant differences in preoperative VAS score, ODI index, BMD value, and Cobb angle between the two groups (*P* > 0.05). At 12 months after surgery, VAS score, ODI index, and Cobb angle decreased, while BMD value increased in both the groups. The VAS score, ODI index, and Cobb angle in the combined group were lower than those in the operation group, while BMD value was higher than that in the operation group, and the difference was significant (*P* < 0.05). According to the differences in VAS score, ODI index, BMD value, and Cobb angle between the two groups one year before and after surgery, the combined use of bisphosphonates had a better promoting effect on pain relief, symptom improvement, and BMD improvement in OVCF patients, as shown in Figure 1:

### 6.3. Comparison of Clinical Data between the Refracture Group and the Nonrefracture Group

Univariate analysis of the clinical data of patients in the refracture group and the nonrefracture group showed that there was no significant difference in the basic data of the two groups, including gender, BMI, and initial fracture site (*P* > 0.05). There were significant differences in age, BMD value, VAS score, and postoperative antiosteoporosis treatment between the two groups, suggesting that age, BMD value, VAS score, and postoperative antiosteoporosis treatment may be related to the occurrence of refracture after vertebroplasty (*P* < 0.05), and the results are shown in Table 2:

### 6.4. Comparison of Surgical Factors between the Refracture Group and the Nonrefracture Group

A single factor analysis was conducted on the operative indicators of the refracture group and the nonrefracture group, and the results showed that there was no statistical significance in the operative indicators, including the amount of bone cement injection, surgical method, and puncture method, between the two groups (*P* > 0.05). The proportion of bone cement diffusion and cement leakage in the refracture group was significantly higher than that in the nonrefracture group, suggesting that poor bone cement diffusion and cement leakage may be related to the occurrence of refracture after vertebroplasty (*P* < 0.05), and the results are shown in Table 3:

### 6.5. Analysis of Influencing Factors of Refracture after Vertebroplasty

The indicators with significant differences in the univariate analysis were used as independent variables (assigned as in the univariate analysis), and the patients were entered into a multifactorial logistic regression analysis with the occurrence of postoperative refracture as the dependent variable. The results showed that BMD, failure of postoperative antiosteoporosis treatment, cement leakage, and poor cement dispersion were independent risk factors for the occurrence of refracture after vertebroplasty in patients with vertebral compression fractures (*P* < 0.05), and the results are shown in Table 4:

## 7. Discussion

Patients with senile and postmenopausal osteoporosis are prone to OVCF due to slight external force due to the loss of vertebral bone mass and significant decline in vertebral strength and bearing capacity. It is the most common complication of postmenopausal osteoporosis (PMOP) and senile osteoporosis, and the main clinical manifestations are pain at the fracture site, limited movement, and kyphotic shape of the spine; Severe cases can lead to disability [16, 17]. Vertebroplasty (PVP/PKP) is an effective minimally invasive procedure for the treatment of OVCF, providing significant pain relief, reducing the incidence of postoperative complications, and allowing patients to get out of bed early and facilitate postoperative recovery [18]. There is no clear understanding of the causes and mechanisms of vertebral refracture after vertebroplasty in patients with OVCF, and it is still controversial whether vertebroplasty causes vertebral refracture.

In this study, 29 of 150 patients with OVCF had secondary vertebral fractures, with a prevalence of 19.33%, a result similar to that reported by Dai et al. [19] reported similar results. At present, the factors affecting secondary vertebral fractures after OVCF vertebroplasty are often divided into two categories: first, the patient's own factors, such as advanced age and preoperative complications of osteoporosis; second, the factors of local biomechanical changes due to vertebral body strengthening, such as the puncture method, cement dispersion, distribution, and leakage in the vertebral body. MA et al. [20] reported no statistically significant difference in the incidence of postoperative secondary vertebral fractures in the conservative treatment group compared with the PVP surgery group, and therefore attributed it to the natural course of osteoporosis, whereas the present study found that patient age was not an independent risk factor for refracture after vertebroplasty, which does not yet support the above conclusion. BMD is an important index for diagnosing osteoporosis and reflecting the efficacy of antiosteoporosis treatment. Low BMD reflects a higher degree of osteoporosis, and the more osteoporotic the vertebral body is, the more likely it is to refracture, which is a risk factor for nonoperative vertebral refracture, and some scholars found that the main risk factor for refracture after vertebroplasty is low BMD through comparative analysis [21]. The BMD of the refractured group in this study was lower than that of the nonrefractured group, and the difference was statistically significant, in line with the above findings, and low BMD was found to be an independent risk factor for vertebral refracture after logistic multifactor analysis. Bone cement leakage is a common complication after vertebroplasty, and a study [22] found that the irreversible damage to soft tissues and bone at the site of cement leakage due to heat setting of bone cement may be a risk factor for refracture after vertebroplasty, and in this study, bone cement leakage was used as an observation index to confirm that bone cement leakage is an associated risk factor for refracture. It was found that good cement dispersion significantly reduced the risk of secondary vertebral fracture after surgery. The results of this study showed that the incidence of refracture was significantly higher in patients with poor cement dispersion than in those with good cement dispersion, and the results of multifactorial analysis showed that poor cement dispersion was an independent risk factor for the occurrence of refracture. This may be related to poor cement dispersion resulting in inadequate axial weight-bearing stresses in the unreinforced region, secondary to recollapse fractures and progressive kyphosis of the reinforced vertebrae, as well as uneven support between the reinforced and unreinforced regions, inconsistent stiffness and postoperative stress concentration transfer to adjacent vertebrae, and overall spinal biomechanical changes [23].

Bisphosphonates are currently the first-line drugs for the clinical treatment of osteoporosis, with long-term clinical data demonstrating their safety and reliability [24, 25]. In this study, we observed the changes of pain, limb function, BMD, and Cobb angle in patients with OVCF after vertebroplasty and standardized antiosteoporosis treatment with bisphosphonates. The results showed that the improvement of ODI index and BMD values at 12 months after surgery were significantly better in the combined group than in the surgical group, indicating that the combined standardized antiosteoporosis drug treatment after surgery can promote the improvement of BMD, relieve pain, and improve quality. We also used whether postoperative antiosteoporosis treatment was standardized as an observation indicator for postoperative refracture in OVCF patients, and the results confirmed that failure to standardize postoperative antiosteoporosis treatment was an independent risk factor for the occurrence of refracture.

In conclusion, in order to avoid recurrent fractures in OVCF patients after surgery, attention should be paid to BMD, whether the patient is taking antiosteoporosis drugs, whether bone cement infiltration occurs, and the dispersion of bone cement. The above factors are the main factors that cause recurrent fractures after PKP and PVP in clinical practice, and should be strictly noted and prevented during the clinical treatment with PKP and PVP in order to improve the clinical prognosis of patients and reduce the occurrence of recurrent fractures.

## Figures and Tables

**Table 1:** Comparison of general data at diagnosis between the surgery group and the combined group.

Factors		Operation group (*n* = 75)	Combined group (*n* = 75)	*t/χ * ^2^)	*P*
Gender	Male	15 (20.00)	17 (22.67)	0.159	0.690
	Female	60 (80.00)	58 (77.33)		
Age (years)		71.80 ± 8.59	72.53 ± 8.41	0.526	0.600

BMI (kg/m^2^)		23.31 ± 4.22	22.89 ± 4.16	0.614	0.540
BMD (T)		−3.62 ± 0.74	−3.59 ± 0.80	0.238	0.812
VAS (分)		8.05 ± 0.74	8.09 ± 0.69	0.342	0.733

Fracture site	≤T10	14 (18.67)	16 (21.33)	0.187	0.911
	T10∼L2	49 (65.33)	48 (64.00)		
	L3∼L5	12 (16.00)	11 (14.67)		

**Table 2:** Comparison of clinical data between the refracture group and the nonrefracture group.

Factors		Refracture group (*n* = 29)	Nonrefracture group (*n* = 121)	*t/χ * ^2^	*P*
Gender	Male	6 (20.69)	26 (21.49)	0.009	0.925
	Female	23 (79.31)	95 (78.51)		
Age (years)		68.10 ± 10.86	73.14 ± 7.54	3.586	0.001
BMI (kg/m^2^)		21.92 ± 5.69	23.38 ± 3.71	1.698	0.092
BMD (T)		−3.97 ± 0.94	−3.52 ± 0.70	2.897	0.004
VAS (points)		7.82 ± 0.97	9.13 ± 0.71	8.273	0.001

Initial fracture site	≤T10	8 (27.58)	22 (18.18)	1.621	0.445
	T10∼L2	16 (55.17)	81 (66.94)		
	L3∼L5	5 (17.24)	18 (14.88)		

Postoperative antiosteoporosis therapy	Yes	12 (41.38)	88 (72.73)	10.345	0.001
	No	17 (58.62)	33 (27.27)		

**Table 3:** Comparison of surgical factors between the refracture group and the nonrefracture group.

Factors		Refracture group (*n* = 29)	Nonrefracture group (*n* = 121)	*t/χ * ^2^	*P*
Bone cement injection volume (mL)		6.02 ± 2.13	5.52 ± 2.76	0.912	0.363

Bone cement leakage	Yes	10 (34.48)	21 (18.36)	4.088	0.043
	No	19 (65.52)	99 (81.82)		

Bone cement	Bad	18 (62.07)	36 (29.75)	10.604	0.001
	Good	11 (37.93)	85 (70.25)		

Surgical method	PVP	18 (62.07)	68 (56.20)	0.330	0.566
	PKP	11 (37.92)	53 (43.80)		

Puncture mode	Unilateral	23 (79.31)	98 (80.99)	0.042	0.837
	Bilateral	6 (20.39)	23 (19.01)		

**Table 4:** Analysis of influencing factors of refracture after vertebroplasty.

Indicators	*B*	SE	Walds	OR	95% CI	*P*
Age	0.312	0.308	1.518	1.366	0.747∼2.498	0.273
BMD	0.511	0.230	11.312	1.667	1.062∼2.616	0.001
Postoperative antiosteoporosis therapy (yes vs. no)	0.078	0.016	19.330	1.081	1.048∼1.116	<0.001
Bone cement leakage (yes vs. no)	0.036	0.008	6.136	1.037	1.021∼1.053	0.009
Bone cement dispersion (good vs. bad)	0.416	0.122	5.246	1.516	1.193∼1.925	0.021

## Data Availability

The data used to support the findings of this study are available from the corresponding author upon request.
